# Transcriptome Analysis Reveals Immune and Antioxidant Defense Mechanisms in the *Eriocheir japonica sinensis* after Exposure to Ammonia

**DOI:** 10.3390/ani14202981

**Published:** 2024-10-16

**Authors:** Xi-Rong Zhu, Ye Jin, Xue Zhang, Qiu-Ning Liu, Bo-Ping Tang

**Affiliations:** 1 Jiangsu Key Laboratory for Bioresources of Saline Soils, Jiangsu Synthetic Innovation Center for Coastal Bio-Agriculture, Jiangsu Provincial Key Laboratory of Coastal Wetland Bioresources and Environmental Protection, School of Wetlands, Yancheng Teachers University, Yancheng 224007, China; 2 College of Biotechnology and Pharmaceutical Engineering, Nanjing University of Technology, Nanjing 210009, China; 3 Key Laboratory of Freshwater Aquatic Genetic Resources, Ministry of Agriculture and Rural Affairs, College of Aquaculture and Life Science, Shanghai Ocean University, Shanghai 201306, China

**Keywords:** *Eriocheir japonica sinensis*, ammonia, immune response, gene expression, lysosomal pathway

## Abstract

As a key species in freshwater aquaculture, *Eriocheir japonica sinensis* was subjected to ammonia stress to assess its impact on the hepatopancreas. A total of 4007 differentially expressed genes (DEGs) were identified between control and treatment groups, comprising 1838 upregulated and 2169 downregulated genes. Following exposure to 300 mg/L of ammonia, the oxidative phosphorylation pathway was activated, while the lysosomal pathway was suppressed, thereby influencing immune functions. Thirteen DEGs from these pathways were further validated via qRT-PCR, revealing gene expression changes of one- to two-fold. Both acid phosphatase (ACP) and alkaline phosphatase (AKP) levels in the hepatopancreas and hemolymph initially increased and then decreased, indicating a disruption in immune functionality. Additionally, alanine transaminase (ALT) and triglyceride (TG) levels were measured, alongside catalase (CAT) activity, total antioxidant capacity (T-AOC), and malondialdehyde (MDA) content, all of which showed an upward trend, signifying oxidative stress and tissue damage. These results offer critical insights into the antioxidant and immune mechanisms of *E. j. sinensis* in ammonia-enriched environments.

## Simple Summary

Ammonia poses a significant environmental hazard in aquaculture, with elevated concentrations exerting various detrimental effects on Chinese mitten crabs (*Eriocheir japonica sinensis*), including reduced growth rates, tissue damage, and increased mortality. This study aimed to investigate gene expression in the midgut of *E. j. sinensis* under ammonia exposure, and contribute to a more profound understanding of the species’ energy metabolism and immune responses under such stress. The findings provide essential baseline data to support the sustainable development of the industry.

## 1. Introduction

*Eriocheir japonica sinensis*, commonly known as the river crab, belongs to Arthropoda, Crustacea, Decapoda, Grapsidae, Varuninae, and Eriocheir [1,2]. This species is a migratory crustacean that grows and matures in freshwater lakes but reproduces in seawater [3]. Known for its high quality and economic value, *E. j. sinensis* plays a pivotal role in aquaculture [4,5]. As an omnivorous crustacean [6], it thrives in freshwater rivers characterized by clear waters and abundant aquatic plants [7].

The aquaculture water environment significantly affects the growth of the Chinese mitten crab, with ammonia and other water quality factors playing a central role [8]. Intensive farming practices often result in the breakdown of protein feed and heterotrophic bacterial metabolites, with decreased dissolved oxygen (DO) levels impairing the nitrifying bacterial activity, thus limiting ammonia degradation. Consequently, ammonia accumulation is a critical factor influencing river crab cultivation. Ammonia is one of the primary environmental stressors in aquaculture, leading to reduced growth rates, tissue damage, and high mortality in *E. j. sinensis* [9].

As water temperatures decrease and gonadal development begins, crabs initiate migration, during which the hepatopancreas supplies essential energy and materials. In crustaceans, the hepatopancreas is vital for digestion and detoxification [10,11]. It serves as the primary site for lipid and carbohydrate metabolism, energy storage and expenditure, and the digestion, absorption, and secretion of nutrients [12]. Previous research has demonstrated that high ammonia concentrations elevate oxygen consumption, impair osmoregulation, affect molting frequency, and damage the hepatopancreas, resulting in increased mortality in crustaceans [9,13]. Several studies have also reported that varying ammonia levels impact the immune function and tissue structure of the hepatopancreas in *E. j. sinensis* [7,14], with similar findings observed in *Portunus trituberculatus* [15] and *Scylla paramamosain* [16]. These insights emphasize the importance of maintaining optimal water conditions during river crab cultivation to support healthy growth.

Given the absence of immunoglobulin-mediated adaptive immunity in fish and crustaceans, their defense mechanisms primarily rely on phagocytosis and encapsulation by hemocytes. Hemocytes are the primary cellular components of the crustacean immune system, responsible for clearing pathogens through adhesion, phagocytosis, and the production of reactive oxygen species (ROS) [17]. Elevated ammonia concentrations reduce blood lymphocyte counts and hemocyanin levels, inhibit hemocyte phagocytic activity, and diminish lysozyme and phenol oxidase activity, ultimately compromising the immune defense system, including non-specific immune cells and hemolymph immune response factors [18,19,20]. The fluctuation of antioxidant indices in hemolymph and the hepatopancreas is an indicator of the overall health status of aquatic species [21]. Numerous studies have explored the effects of high ammonia exposure on aquatic organisms, including *Marsupenaeus japonicas* [13], *Litopenaeus vannamei* [22], *Dicentrarchus labrax* [23,24], *Macrobrachium acanthurus* [25], *Portunus trituberculatus*, and *E. j. sinensis* [26]. However, molecular studies examining the hepatopancreas and hemolymph of *E. j. sinensis* under ammonia stress remain limited. This research holds substantial value in elucidating the autoimmune mechanisms of *E. j. sinensis* and contributing to its sustainable aquaculture, providing essential data for ensuring the species’ healthy cultivation.

## 2. Materials and Methods

### 2.1. Laboratory Animals and Temporary Management

The experimental animal is *E. j. sinensis*, which was purchased from a market in Yancheng, Jiangsu Province, China. Twenty Chinese mitten crabs were randomly selected and dried with filter paper to measure their body weight and length. The average wet weight and length was 44.96 ± 4.84 g and 4.5 ± 0.27 cm, respectively. Before the start of the experiment, the Chinese mitten crabs were temporarily raised in a plastic bucket (measuring 860 mm in length, 630 mm in width, and 480 mm in height) for 15 days. During the temporary cultivation period, the water was changed daily. The water used in the experiment was tap water that had been left to stand for 24 h. The natural light period was maintained, they were fed at a fixed time every day using TONGWEI FEED from China, and they were provided with sufficient oxygen. The health of the Chinese mitten crabs was observed daily, and any dead crabs were removed promptly.

### 2.2. Acute Ammonia Stress Experiment

Firstly, 10 ± 0.01 g of NH_4_Cl (Sinopharm Chemical Reagent Co., Ltd., Shanghai, China) were weighed. The fully aerated tap water was then dissolved in a 500 mL beaker, and the volume was adjusted in a 1 L capacity bottle to create a 10 g/L solution. Based on pre-experiment, the 96 h semi-lethal concentration of NH_4_Cl was calculated, determining the safe concentration to be 249.13 mg/L. At this concentration, ammonia levels were set to 0, 100 mg/L, 300 mg/L and 600 mg/L. One hundred and eighty Chinese mitten crabs of similar size, with normal, healthy, lively bodies, and intact appendages were selected. They were divided into four groups of 45 crabs each. These 45 crabs were placed into three white plastic pots (46 cm in length, 32 cm in width, and 15 cm in height) and exposed to varying concentrations of ammonia for 96 h. Throughout the experiment, the solution in the containers was replaced daily with a new solution containing the same concentration of ammonium chloride. Feed was given at scheduled times, and sufficient oxygen was provided. The health of the Chinese mitten crabs was monitored daily, and any dead crabs were removed promptly.

### 2.3. Sampling and Preservation

The duration of the ammonia stress experiment was 4 d, with samples taken at 24 h, 48 h, 72 h, and 96 h. Six Chinese mitten crabs were randomly selected from each group for sampling. After removing the river crab, hemolymph was collected from the base of the third step, and the extracted hemolymph was immediately mixed with anticoagulant using a 1 mL syringe in a 1:1 ratio. The anticoagulant consisted of NaCl 0.14 mol/L, citric acid 0.03 mol/L, trisodium citrate 0.026 mol/L, glucose 0.1 mol/L, EDTA· Na_2_ 0.01 mol/L, with a pH of 4.6, and sterilized at 121.3 °C for 20 min. The mixture was then centrifuged at 5000× *g* for 10 min at 4 °C to separate blood cells and blood cell suspensions, which were stored in a cryogenic refrigerator at −80 °C. Blood cells were used for mRNA extraction, while blood cell suspensions were utilized for enzyme activity tests. The hepatopancreas of the crab was promptly removed and stored in a cryogenic refrigerator at −80 °C for further mRNA and enzyme activity experiments, and ensure three biological repeats.

### 2.4. Histopathological Section

After 96 h of ammonia stress, three hepatopancreases of *E. j. sinensis* were randomly selected from the control group and the 300 mg/L concentration group, and fixed with 4% formaldehyde for 24 h. The paraffin blocks were cut into 5 μm slices using a slicer (Leica CM1950, Nussloch, Germany), stained with hematoxylin and eosin, fixed with neutral gum, observed under a microscope (Motic, Xiamen, China), and tissue section photos were collected.

### 2.5. TUNEL Assay

The fresh hepatopancreatic tissue was sliced, then dried and covered with protease K working solution before being placed in an incubator at 37 °C for 22 min. The tissue was washed and decolorized three times for 5 min each using PBS. To create the TUNEL detection solution, TDT enzyme, dUTP, and buffer were mixed at a ratio of 1:5:50 and incubated at 37 °C for 2 h. After incubation, the slices were washed with PBS three times for 5 min each, then DAPI staining solution was added, and they were then incubated in the dark for 10 min. The sections were washed three times for 5 min each with PBS and then sealed with anti-fluorescence quenching before being dried. Finally, the sections were observed under a fluorescence microscope (Leica, Germany) and the images were collected [27].

### 2.6. Determination of Nonspecific Enzyme Activity

#### 2.6.1. Sample Pretreatment

Three hepatopancreatic tissues of *E. j. sinensis* were randomly selected from each group after 96 h of ammonia stress. An appropriate amount of hepatopancreas tissue was weighed, and physiological saline was added according to the ratio of weight (g) to volume (mL) of 1:9. The tissue was then mechanically homogenized under ice water bath conditions to create a 10% tissue homogenate. The homogenate was centrifuged at 4 °C and 2500 rpm for 10 min, and the supernatant was taken for detection. The hemolymph was placed on ice to await testing.

#### 2.6.2. Biochemical Analysis

The acid phosphatase (ACP), alkaline phosphatase (AKP), alanine aminotransferase (ALT), triglyceride (TG), catalase (CAT), and total antioxidant capacity (T-AOC) in the hepatopancreatic tissue and hemolymph of *E. j. sinensis* were determined using the corresponding kit (Jiancheng, Ltd., Nanjing, China) according to the manufacturer’s instructions.

### 2.7. RNA Extraction, Library Construction, and Illumina Sequencing

According to the manufacturer’s instructions, each hepatopancreas tissue sample was ground with a mortar, and then total RNA was extracted using Trizol reagent (Vazyme, Nanjing, China). The RNA was then analyzed using 1% agarose gel electrophoresis for detection. The isolated RNA was further examined for purity, concentration, and integrity using a Nano Drop 2000 spectrophotometer (Thermo Scientific, Waltham, MA, USA) and an Agient2100/Lab Chip GX bioanalyzer (Agilent, Santa Clara, CA, USA).

Once the samples were qualified, three samples were synthesized from three hepatopancreatic tissues in the treatment group to construct the cDNA library, and then three samples were synthesized from three hepatopancreatic tissues with a concentration of 300 mg/L to construct the cDNA library. The process involved separating mRNA from total RNA using magnetic beads, fragmenting the mRNA with fragmentation buffer, transcribing the mRNA into cDNA using reverse transcriptase, synthesizing the first and second cDNA chains, purifying the cDNA, adding appropriate connectors A-tail and sequencing linker, selecting the fragment size with AM Pure XP beads, and amplifying and enriching the cDNA through polymerase chain reaction to create the cDNA library. After passing the quality inspection, the cDNA library was sequenced in PE150 format using the Illumina Nova Seq 6000 sequencing platform (Illumina, San Diego, CA, USA).

### 2.8. Transcriptome Analysis and Functional Annotation of Unigenes

The cDNA library was sequenced using the Illumina transcriptome sequencing platform, resulting in the generation of a large amount of raw data. After undergoing a series of quality control procedures, high-quality Clean Data were obtained and provided in FASTQ format to ensure the accuracy of subsequent analyses. The specific filtering methods employed included ensuring that the Reads were of high quality and implementing strict data quality control measures. This involved removing reads containing adapters and filtering out low-quality reads (including those with an N ratio greater than 10% and reads where the Q value was less than or equal to 10 accounting for more than 50% of the read) [28].

Reads obtained by RNA-seq were short fragments of randomly interrupted mRNA, and the obtained high-quality Clean Reads were compared with the reference genome to determine which genes transcribe these Reads. We used the designated as a reference for sequence alignment and subsequent analysis. Version information of reference genome: *E. j. sinensis*. ASM2467909v1.genome.fa. We used the HISAT2 [29] software (Version 2.2.1) to quickly and accurately compare Clean Reads with the reference genome, and obtained the positioning information of Reads on the reference genome. Then, the reads on the database were assembled using StringTie comparison, and the transcriptome was reconstructed for subsequent analysis.

The output of RNA-seq was sequence fragments (Reads), and the expression level of the gene was calculated according to the number (Count) of each transcript compared with the sequencing reads. We then normalized the number of Mapped Reads and transcript length in the sample. StringTie was used to standardize using the maximum flow algorithm and FPKM [30] (Fragments Per Kilobase of transcript per Million fragments mapped) as an index to measure the expression level of transcripts or genes.

Gene function annotation was carried out using various databases including the National Biotechnology Information Center (NCBI) Non-redundant Protein (NR) database, which links nucleic acid data with protein data; the NCBI Non-redundant Nucleotide Sequence (NT) database; the Swiss-Prot database [31]; the Protein family (PFAM) database [32]; the Protein linear cluster/eukaryote cluster (COG/KOG) database [33]; the Gene ontology (GO) database [34]; and the Kyoto Encyclopedia of Genes and Genomes (KEGG) [35]. Blast2GO software (Version 4.5.1) was utilized to assign GO annotations [36].

### 2.9. Identification of Differentially Expressed Genes and Gene Enrichment Analysis

By utilizing the TMs method to calculate the expression of a single gene and estimating the gene expression level of each sample, differentially expressed genes (DEGs) between the two libraries were identified. The DESeqR package (1.12.10) was used to analyze the differentially expressed genes in the two libraries [37]. The Count values of genes in different samples were used to screen for differentially expressed genes using differential analysis software, with the screening criteria of a differential multiple being log2(FC) ≥ 1 and *p* ≤ 0.05.

The DEGs identified above were then analyzed using GOSeqR [38] and KOBAS [39] successively for GO function enrichment and KEGG pathway analysis. Gene enrichment analysis is a method of analyzing gene expression information, which primarily includes gene function enrichment (GO enrichment analysis) and metabolic pathway enrichment analysis (KEGG enrichment analysis).

### 2.10. Quantitative Real-Time PCR (qRT-PCR)

According to the data obtained from the sequencing of the transcriptome previously submitted for inspection, the RNA extracted from hepatopancreas samples was first reverse transcribed into cDNA using a reverse transcription kit (Vazyme, Nanjing, China). The cDNA, along with the β-actin gene as a reference gene, was then used as a template [40]. Seven differential genes were randomly selected from the sequencing results, and the qRT-PCR method was conducted following established protocols [41]. Gene-specific primers were designed using Primer Premier 5.0 (Table 1) and synthesized by biological companies (GENERAL BIOL, Chuzhou, Anhui, China). The qRT-PCR reaction was performed in a 10 µL volume, containing 5 µL of 2 × SYBR Green qPCR Mix, 0.5 µL of each upstream and downstream primers, 2 µL of cDNA template, and 2 µL of sterilized water. The cycling conditions were as follows: 95 °C for 15 s, 55 °C for 20 s, and 72 °C for 30 s, for a total of 40 cycles. All reactions were performed in triplicate. Gene expression levels were quantified using the 2^−ΔΔCT^ method [42].

### 2.11. Data Processing

The results are presented as means ± SE. The normality of the experimental data was assessed using SPSS Statistics (Version 20), and differences between groups were evaluated through independent sample *t*-tests. Statistical significance is indicated by *p*-values, where *p* < 0.001 denotes extremely significant differences, *p* < 0.01 indicates highly significant differences, and *p* < 0.05 indicates significant differences. Graphs were created using GraphPad Prism 9, with data analysis and statistical evaluations performed using Excel and IBM SPSS Statistics 23.

## 3. Results and Discussion

### 3.1. Effect of Ammonia on Histopathology of Hepatopancreas of E. j. sinensis

**Figure 1:**
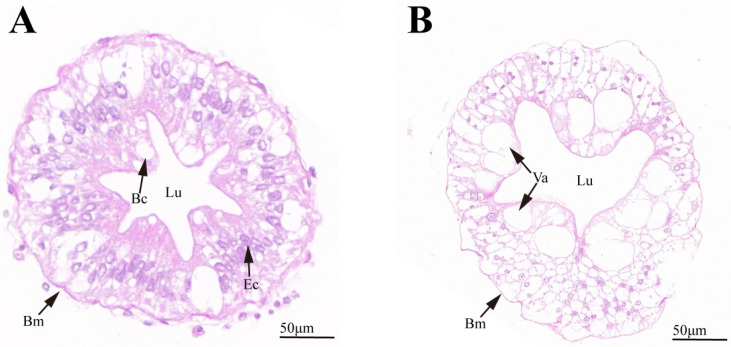
HE staining of hepatopancreatic tissue structure of *E. j. sinensis* under ammonia stress. (**A**), control group; (**B**), medium-concentration stress group (300 mg/L); Bm, basement membrane; Bc, secretory cell; Ec, embryonic cell; Va, vacuole; Lu, lumen.

The histological results of the hepatopancreatic tissue sections are presented in Figure 1. In the control group, the hepatopancreas of *E. j. sinensis* exhibited a well-preserved structure, with normal hepatic tubules, clear and well-defined boundaries, and an intact basement membrane. The epithelial cells were uniformly arranged, and the epithelium adhered tightly to the underlying cells. Vacuoles were minimal, and the lumen displayed a clear, star-shaped structure. In contrast, in the 300 mg/L ammonia stress group, significant tissue damage was observed. The hepatic tubule morphology was disrupted, the basement membrane was compromised, and the epithelium became detached from the epithelial cells. Both the number and size of vacuoles increased markedly, the stellate structure of the lumen disappeared, and fragmented particulate matter was visible within the lumen. These results indicate that ammonia stress induced notable hepatopancreatic damage in *E. j. sinensis*, impairing the physiological functions of the organ.

Similar to other aquatic species, the hepatopancreas in *E. j. sinensis*, as a major immune organ, plays a critical role in detoxification and immune responses. It primarily consists of B cells (secretory cells), E cells (embryonic cells), and R cells (storage cells) [43]. Studies have demonstrated that the hepatopancreas is highly sensitive to external toxins, and the extent of tissue damage often correlates with the severity of toxin exposure. The greater the toxicity, the more severe the damage to hepatopancreatic tissue structure [44]. In this experiment, the hepatopancreas sustained considerable injury. Similar histopathological alterations have been reported by Wang et al. [45], who observed damage in the hepatopancreas of *Penaeus vannamei* exposed to aflatoxin B1. Additionally, research on *Carassius auratus* has shown that ammonia stress can cause hepatocyte alterations, such as vacuolization, nuclear shrinkage and dissolution, and blurred cell membrane boundaries [43].

### 3.2. Effects of Ammonia Stress on Nonspecific Enzyme Activities in Hepatopancreas of E. j. sinensis

**Figure 2:**
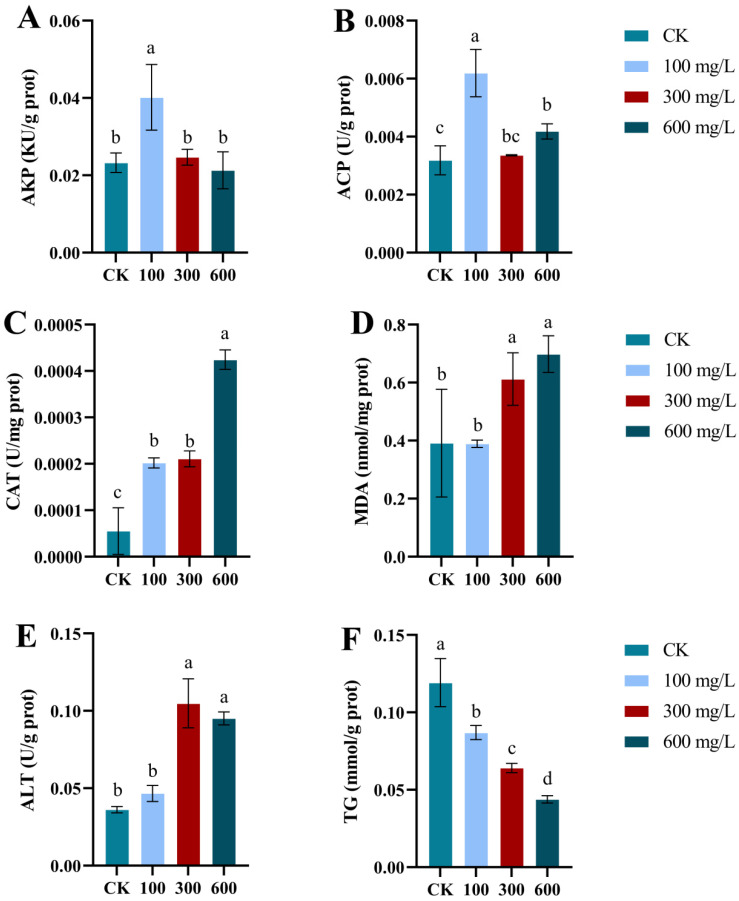
Effects of ammonia stress on non-specific enzyme activity in *E. j. sinensis*. (**A**) AKP in hepatopancreas; (**B**) ACP in hepatopancreas; (**C**) CAT in hepatopancreas; (**D**) MDA in hepatopancreas; (**E**) ALT in hepatopancreas; (**F**) TG in hepatopancreas. Different letters (a, b, c, d) indicate significant differences (*p* < 0.05).

Figure 2 illustrates the impact of ammonia exposure on the activities of alkaline phosphatase (AKP, A) and acid phosphatase (ACP, B) in the hepatopancreas of *E. j. sinensis*. Compared with the control group, the activities of both enzymes initially increased, followed by a decline, and then rose again. However, at a concentration of 300 mg/L ammonia, a significant reduction in enzyme activity was observed (*p* < 0.05). As ammonia concentration increased, catalase (CAT, C) activity in the hepatopancreas also showed a dose-dependent increase, with significantly higher activity in the treatment group compared to the control (*p* < 0.05). As ammonia concentration increased, malondialdehyde (MDA, D) levels rose in a concentration-dependent manner. Alanine aminotransferase (ALT, E) activity in the hepatopancreas of *E. j. sinensis* gradually increased after 96 h of ammonia stress, peaking at 300 mg/L, before declining. Nevertheless, ALT activity remained higher than in the control group. In Figure 2F, triglyceride (TG) levels in the hepatopancreas of *E. j. sinensis* decreased progressively over 96 h of ammonia stress and were significantly lower than those in the control group (*p* < 0.05).

AKP and ACP play vital roles in the non-specific immune defense of crustaceans, participating in hydrolysis processes by transferring and metabolizing phosphate groups to eliminate foreign substances and maintain health [46]. Molina et al. demonstrated that ACP and AKP activities in the liver of *Oreochromis mossambicus* significantly increased after exposure to *Microcystis aeruginosa* for 21 days [47]. Similarly, Qin et al. reported that ACP activity in the hemolymph of *Sinopotamon henanensis* initially rose and then decreased under acute cadmium exposure [48]. The fluctuations in AKP and ACP activities observed in this study suggest that ammonia stress could compromise the non-specific immune defenses of *E. j. sinensis*. We further hypothesize that the abnormal activity levels of these enzymes may indicate hepatopancreatic damage and dysfunction.

CAT is a crucial enzyme in cellular antioxidant defense mechanisms and plays a vital role in the biological defense system. Predominantly found in peroxisomes and microsomes, CAT decomposes hydrogen peroxide (H_2_O_2_) into molecular oxygen and water, preventing H_2_O_2_ from reacting with O^2−^ to form harmful hydroxyl radicals (·OH). This action protects against the peroxidation of unsaturated fatty acids in biological membranes, reducing the fluidity of the cell membrane and preventing damage to nucleic acids and chromosomes [44,49]. Similar findings have been observed in other aquatic species. For example, in *Pelteobagrus fulvidraco*, hepatic CAT activity increased after 96 h of ammonia stress [50], and after 24 h of ammonia exposure, liver CAT activity also showed an increase [51]. These results align with those of the present study, where CAT activity in the hepatopancreas of *E. j. sinensis* increased following ammonia exposure. This suggests that changes in ammonia concentration in the aquatic environment induce oxidative stress in aquatic organisms, leading to excessive production or accumulation of ROS, which can cause cellular damage. In response, the organism’s antioxidant defense system is activated, including an increase in antioxidant enzyme activity such as CAT.

MDA is a lipid peroxidation product that serves as an indicator of oxidative damage in organisms [52]. The findings of this study indicate that ammonia stress elevates MDA levels in the hepatopancreas of *E. j. sinensis*. Increased MDA content suggests that the organism is under oxidative stress, and the antioxidant system is unable to adequately remove excess ROS, resulting in severe lipid membrane peroxidation and subsequent tissue and cellular damage [53]. Similar observations have been made in other aquatic studies. For instance, Li et al. demonstrated that MDA content in the liver of *Pelteobagrus fulvidraco* increased after both 96 h and 56 days of ammonia stress [54,55]. These results are consistent with the upward trend in MDA levels observed in *E. j. sinensis* under ammonia stress in this study, all of which suggest that environmental stress leads to elevated MDA levels in aquatic organisms, reflecting oxidative stress and tissue damage.

ALT primarily resides in the cytoplasm of hepatocytes. As a key biomarker for liver function, ALT levels are instrumental in preliminarily assessing hepatopancreatic damage and the extent of liver dysfunction [56]. A marked increase in ALT levels is indicative of significant hepatopancreatic injury and heightened cellular permeability [57]. In this experiment, ALT levels increased notably following 96 h of ammonia exposure at a concentration of 300 mg/L, suggesting that ammonia stress may lead to hepatopancreatic tissue damage and increased membrane permeability. This finding aligns with previous studies, where ammonia exposure elevated ALT levels in *Paramud* and *Cyprinus carpio* [58,59].

Lipids are a key energy source for *E. j. sinensis*. In a study by Wang et al., diets supplemented with FDPA freeze-dried powder significantly reduced TG levels in the hepatopancreas of *Penaeus vannamei* compared to a control diet [60]. Similarly, Xu et al. demonstrated that stimulating mouse adipocytes with endoplasmic reticulum stress-inducing agents inhibited TG decomposition [61]. It is hypothesized that, in response to increasing ammonia concentrations, the organism accelerates TG breakdown to generate more ATP, compensating for the hepatopancreatic damage caused by ammonia stress.

### 3.3. Detection of Apoptosis in TUNEL Cells

**Figure 3:**
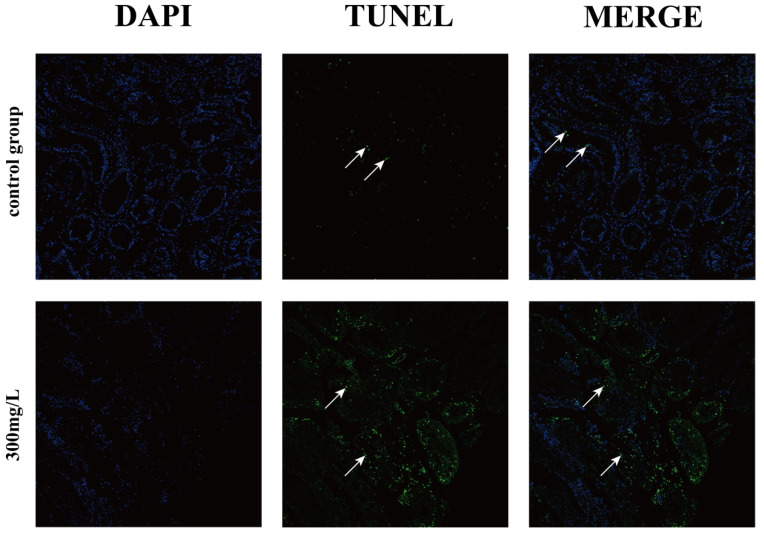
Results of cell apoptosis detection. Arrows represent apoptotic cells.

As depicted in Figure 3, the control group exhibited a small number of apoptotic cells in the hepatopancreas, with an apoptosis rate of 3.33%. In contrast, the group exposed to 300 mg/L ammonia showed a significantly higher proportion of apoptotic cells, with an apoptosis rate of 8.70%, representing an increase of 5.37%. These results indicate a substantial rise in apoptosis compared to the control group. It is hypothesized that ammonia stress at 300 mg/L may impair antioxidant enzyme function and suppress the antioxidant defense system in *E. j. sinensis*, leading to elevated levels of the lipid peroxidation product MDA. This increase in MDA is believed to drive a significant rise in apoptosis, thereby causing damage to hepatopancreatic tissue and overall organ function.

TdT-mediated dUTP nick-end labeling (TUNEL) is an immunodetection method that visually displays the distribution of apoptotic cells within tissue and provides an intuitive means of quantifying cell apoptosis. Based on tissue dynamics research, the body has a natural mechanism for recognizing and eliminating apoptotic cells. When large numbers of apoptotic cells are present, phagocytes function to remove them in a timely manner, thus maintaining a certain level of regulation [44]. However, when this mechanism is compromised, apoptotic cells may accumulate within the body. Zou et al. demonstrated that exposure to perfluorooctanoic acid (PFOA) during pregnancy significantly induced apoptosis in the lung tissue cells of pregnant rats, with an apoptosis rate approaching 40% [62]. Similarly, cadmium exposure triggered the expression of the *caspase-3A* gene in carp, leading to apoptosis in liver cells [63]. Research by Rebecca and David further revealed that chemical and physical stress could induce apoptosis in sea urchin embryos [44], while Chang et al. found that temperature stress could lead to apoptosis in *Litopenaeus vannamei* [64]. Ammonia exposure induced oxidative stress (decreased SOD activity and increased MDA content) in *Litopenaeus vannamei*, which led to apoptosis of the hepatopancreas [65]. These results underscore the role of environmental stressors in causing oxidative stress and apoptosis, paralleling the observed effects of ammonia stress in *E. j. sinensis* in this study.

### 3.4. Effect of Ammonia Stress on Hemolymph Nonspecific Enzyme Activity of E. j. sinensis

**Figure 4:**
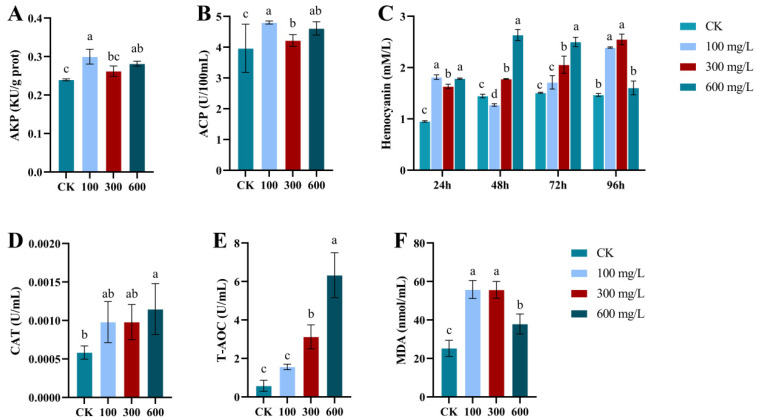
Effect of ammonia stress on non-specific enzyme activity in *E. j. sinensis*. (**A**) AKP in hemolymph; (**B**) ACP in hemolymph; (**C**) hemocyanin content; (**D**) CAT in hemolymph; (**E**) T-AOC in hemolymph; (**F**) MDA in hemolymph. Different letters (a, b, c, d) indicate significant differences (*p* < 0.05).

Figure 4 shows the effect of ammonia exposure on the activities of AKP (A) and ACP (B) in the hemolymph of *E. j. sinensis*. Initially, AKP and ACP activities increased, then declined, followed by another rise. At a concentration of 300 mg/L ammonia, enzyme activity significantly decreased (*p* < 0.05).

Figure 4C illustrates the effect of ammonia stress on hemocyanin content in *E. j. sinensis*. Following 24 h of ammonia exposure, hemocyanin levels initially increased, then decreased, followed by another increase, all of which were significantly higher than those in the control group (*p* < 0.05). After 48 h of stress, hemocyanin levels first declined and then rose again, with significant differences observed across all concentrations. Notably, the 100 mg/L concentration was significantly lower than the control group. By the 72-h mark, hemocyanin levels in the treatment group were significantly higher than those in the control (*p* < 0.05), with an initial rise followed by a decline after 96 h of ammonia stress, once again significantly higher than the control. After 96 h, hemocyanin content first increased and then decreased, with a notable decrease at high ammonia concentrations.

In contrast to the control group, hemolymph CAT activity increased progressively with ammonia concentration, with CAT activities at 100 mg/L and 300 mg/L reaching the same level (Figure 4D), both significantly higher than the control (*p* < 0.05). Similarly, total antioxidant capacity (T-AOC) showed a concentration-dependent increase (Figure 4E), with activities at 300 mg/L and 600 mg/L also significantly higher than the control (*p* < 0.05). This suggests that increasing ammonia concentrations activate the antioxidant mechanisms in *E. j. sinensis*. Additionally, MDA levels in the hemolymph initially increased and then decreased with rising ammonia concentrations (Figure 4F), with the treatment group exhibiting significantly higher levels than the control (*p* < 0.05). MDA content peaked at 300 mg/L.

Foreign substances like ammonia can induce stress and damage to the hepatopancreas, subsequently inhibiting the release of AKP and ACP in hemolymph. Previous research has indicated that ammonia has a detrimental effect on the immune systems of aquatic organisms [66,67]. Tian et al. found that under salinity stress, ACP and AKP activities initially increased and then decreased, a trend that mirrors the low-concentration findings in this study [67]. These results suggest that when crustaceans encounter environmental fluctuations, their bodies detect external stressors and activate immune defense mechanisms, leading to an early rise in hemolymph ACP and AKP activities. However, as the stress persists and exceeds the tolerance levels of the crustaceans, it results in damage to the hepatopancreas, thereby inhibiting the release of these enzymes in hemolymph. Consequently, AKP and ACP activities decline during the later stages of stress. In this study, the increased AKP and ACP activities in *E. j. sinensis* under high-concentration ammonia stress could reflect the organism’s attempt to bolster immune defenses in response to injury and stress. This elevated enzyme activity in the hemolymph might represent the organism’s effort to restore and enhance immune function, compensating for the earlier immune decline caused by hepatopancreatic damage.

Hemocyanin is a unique respiratory protein found in the hemolymph of arthropods. As a multifunctional protein, hemocyanin contains Cu^2+^ as a cofactor and exhibits various functions, including antibacterial, antiviral, and phenoloxidase activity. It plays a critical role in the immune system and participates in other physiological and biochemical processes [68]. Numerous studies have demonstrated that hemocyanin expression increases in crustaceans under environmental stress [40], aligning with the findings in this study, where the hemocyanin content in *E. j. sinensis* initially increased after ammonia stress. This suggests that hemocyanin is likely involved in the response of crustaceans to environmental challenges. However, unlike other studies, the hemocyanin content in *E. j. sinensis* showed a declining trend later in the experiment. This decline may be attributed to the significant physiological stress experienced by *E. j. sinensis*, which could have impacted the stability or synthesis of hemocyanin, accelerating its breakdown and thereby reducing its overall content. Furthermore, medium and high concentrations of ammonia may interfere with the normal function of hemocyanin in oxygen transport and immune defense, prompting the organism to adjust hemocyanin levels in response to this adverse effect.

Previous studies, such as Liang et al., have reported an increase in MDA concentration in *Penaeus vannamei* following exposure to high levels of ambient ammonia. Similarly, Huang et al. observed a significant rise in MDA content in the hemolymph of river crabs under high concentrations of avermectin treatment [69]. These findings are consistent with the results of this study, wherein elevated MDA levels indicate that the organism is under oxidative stress, and its antioxidant system is unable to effectively eliminate excessive ROS, leading to severe lipid membrane peroxidation and subsequent tissue and cellular damage. The higher the MDA content, the greater the oxidative stress experienced. MDA can also compromise the crab’s antioxidant defense system, disrupting the function of antioxidant enzymes and causing further damage [53]. However, after exposure to high concentrations of ammonia, a decline in MDA levels was observed. This may be due to the activation of self-regulatory mechanisms in the later stages of ammonia stress, aimed at mitigating oxidative stress damage to tissues and cells.

### 3.5. Construction and Analysis of Transcriptome Library

In this study, the control group generated 20,791,412, 19,925,204, and 23,122,701 clean reads, corresponding to 6,226,997,816, 5,968,030,201, and 6,924,535,291 nucleotides, respectively. Meanwhile, the treatment groups produced 20,552,156, 20,324,902, and 20,327,981 clean reads, corresponding to 6,151,444,046, 6,084,546,375, and 6,083,545,895 nucleotides, respectively (Table 2).

### 3.6. Functional Annotations and Classification

To obtain comprehensive functional gene information, 76,091 genes were annotated across seven databases: NR, SwissProt, PFAM, KOG, GO, COG, and KEGG. The number of genes annotated in each database was as follows: NR: 14,602 (19.2%); SwissProt: 7944 (10.4%); PFAM: 14,805 (19.5%); KOG: 10,175 (13.4%); GO: 14,180 (18.6%); COG: 4096 (5.4%); and KEGG: 10,289 (13.5%) (Table 3).

### 3.7. Identification and Analysis of DEGs

**Figure 5:**
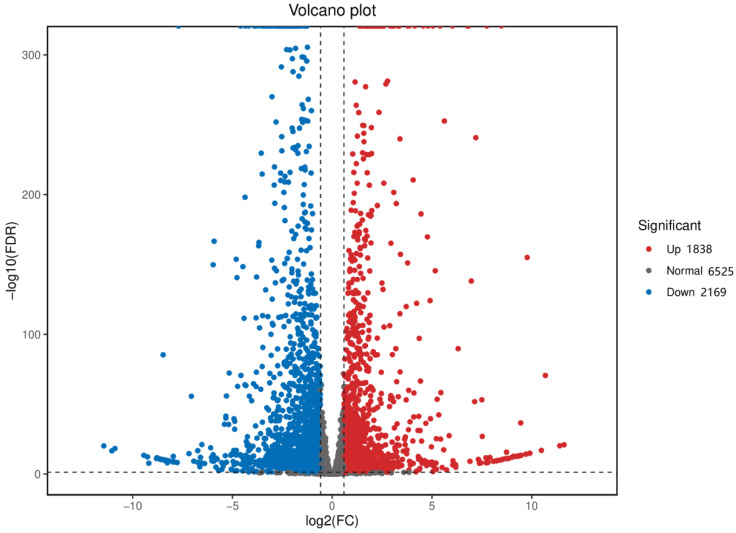
Volcano plot of differentially expressed genes (DEGs) from the transcriptomes of the control and treatment groups. For each unigene, the ratio of expression levels was plotted against the -log error rate. The *x*-axis represents the fold-change between the 300 mg/L ammonia and PBS control groups, and the *y*-axis indicates the significance of differential expression. Gray dots indicate genes with no significant difference, while red and blue dots indicate upregulated and downregulated unigenes, respectively (q-value < 0.05 and |log_2_ (fold-change)| > 1).

A volcano plot was generated based on these results, showing 4007 differentially expressed genes (DEGs) between the control and treatment groups, with 1838 upregulated and 2169 downregulated genes (Figure 5). In the volcano plot, red dots represent upregulated genes, blue dots represent downregulated genes, and gray dots represent genes with no significant differential expression [70].

**Figure 6:**
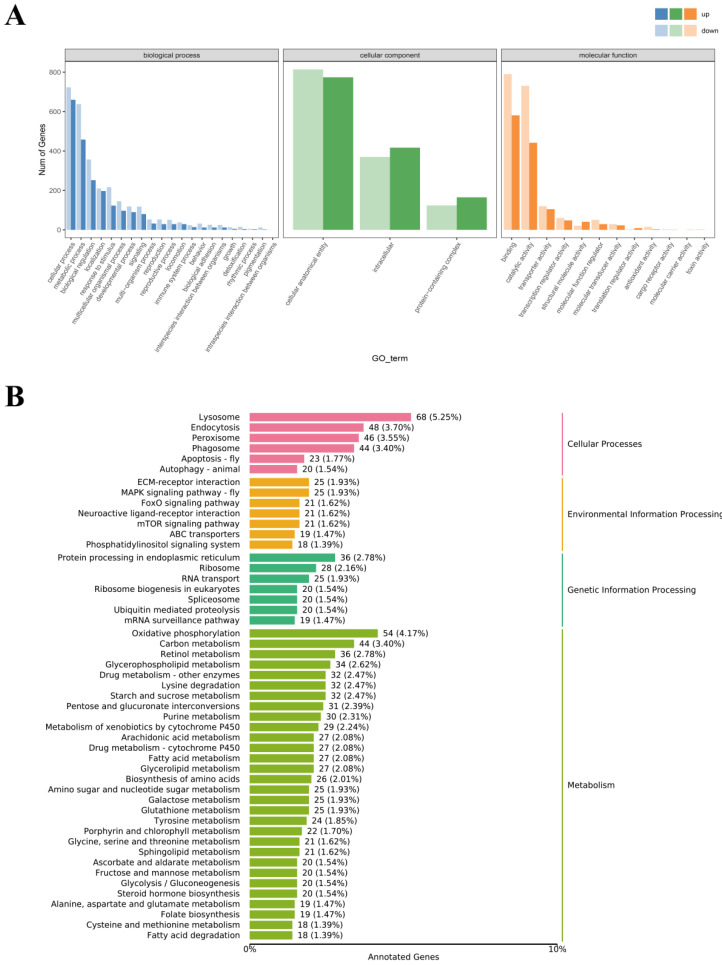
DEG enrichment analysis: (**A**) GO classification of unigenes from the hepatopancreas transcriptome of *E. j. sinensis*. Each annotated sequence is assigned to at least one GO term under the categories of biological process, cellular component, or molecular function. (**B**) KEGG pathway analysis of DEGs in *E. j. sinensis* hepatopancreas.

The GO classification categorizes gene functions into three primary domains: cellular components (CC), molecular functions (MF), and biological processes (BP). Using the Blast program, 2864 DEGs were classified into these three main categories and further subdivided into 36 additional groups. Within the BP category, the predominant genes were associated with cellular processes, metabolic processes, and biological regulation. For MF, binding and catalytic activity constituted the largest groups, while in CC, most genes were linked to cellular anatomical entities and intracellular structures (Figure 6A).

The KEGG database provides a framework linking genomic and functional information. To further explore the role of DEGs in the immune response of *E. j. sinensis* to ammonia, KEGG was employed to analyze the enrichment of relevant pathways. In this study, 2608 DEGs were mapped to 249 pathways, which were grouped into four distinct categories (Figure 6B). In the metabolism category, oxidative phosphorylation (54 DEGs) and carbon metabolism (44 DEGs) were most prominent. For genetic information processing, protein processing in the endoplasmic reticulum (36 DEGs) was the most enriched. The environmental information processing category highlighted ECM-receptor interaction and MAPK signaling pathway-fly (25 DEGs). Lastly, the cellular processes category was dominated by lysosome (68 DEGs) and endocytosis (48 DEGs).

**Figure 7:**
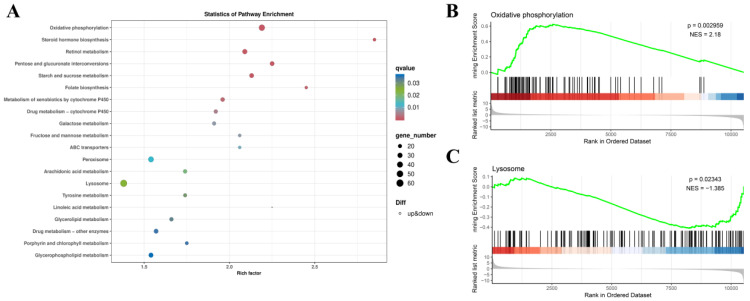
KEGG enrichment bubble diagram (**A**) and GSEA results (**B**,**C**) of DEG analysis.

The results of KEGG pathway enrichment are illustrated through the KEGG bubble diagram (Figure 7A), displaying the top 20 pathways with the lowest q-values. Notably, the oxidative phosphorylation (ko00190) and lysosome (ko04142) pathways were of particular interest. Gene set enrichment analysis (GSEA) [71] revealed that oxidative phosphorylation was upregulated (Figure 7B), while the lysosome pathway was downregulated (Figure 7C).

**Figure 8:**
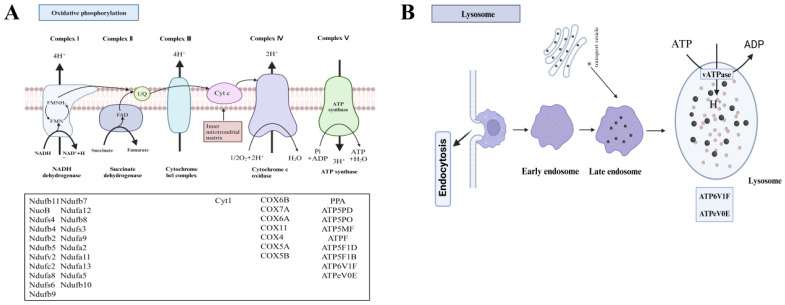
(**A**) Oxidative phosphorylation pathway; (**B**) Lysosome pathway.

Oxidative phosphorylation represents the coupling of ADP and inorganic phosphate to synthesize ATP, driven by the energy released during cellular oxidation through the respiratory chain. This process constitutes the final stage of cellular respiration [72]. Mitochondria, often termed the “powerhouses” of eukaryotic cells, rely heavily on oxidative phosphorylation as a key metabolic function. Among crustaceans, dysfunction in any of the five primary protein complexes (I–IV) is often lethal [73,74] or, at the very least, severely compromises environmental adaptability due to their critical role in energy production [75]. The oxidative phosphorylation pathway is located in the inner mitochondrial membrane and consists of the electron transport chain and ATP synthase, including five complexes: NADH dehydrogenase, succinate dehydrogenase, cytochrome c reductase, cytochrome c oxidase, and ATP synthase (Figure 8A).

Crustaceans, including *E. j. sinensis*, lack an adaptive immune system and thus predominantly rely on innate immunity to withstand environmental challenges. Innate immunity comprises both cellular and humoral defenses, the latter involving the production of ROS [76]. While low ROS levels exhibit antibacterial effects, elevated ROS levels can damage lipids, proteins, and DNA [77,78]. ROS generation primarily occurs in the mitochondrial oxidative phosphorylation pathway [79], with some ROS production involving cytochrome c oxidase, influencing both mitochondrial oxidative phosphorylation capacity and innate immune responses [80,81].

The lysosomal pathway plays a pivotal role in material metabolism, energy regulation, immune defense, and cellular homeostasis [68,82]. During environmental stress, endocytosis allows the organism to engulf pathogens, transporting them to early endosomes, which subsequently mature into late endosomes. These then fuse with phagosomes and autophagosomes to enter lysosomes for degradation (Figure 8B). Throughout this process, ATP and V-ATPase, provided by metabolic pathways, are indispensable. Studies indicate that V-ATPase hydrolyzes one ATP for every two protons transferred [83]. KEGG pathway enrichment analysis indicated an upregulation of the oxidative phosphorylation pathway, a result corroborated by GSEA analysis (Figure 7B). Oxidative phosphorylation converts the chemical energy of organic molecules into ATP. These results suggest an increase in ATP synthesis, potentially enhancing the proton transfer efficiency of V-ATPase to phagocytes. However, despite this, the lysosomal pathway was downregulated. This downregulation could be attributed to oxidative damage caused by ammonia stress, impairing other metabolic pathways and reducing lysosomal energy supply, thereby compromising immune function.

### 3.8. qRT-PCR Results

**Figure 9:**
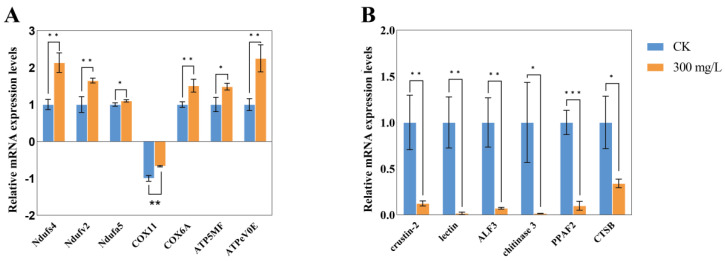
Validation of RNA-seq data by qRT-PCR. (**A**) Relative expression profiles of seven selected oxidative phosphorylation pathway genes in the hepatopancreas after ammonia stress compared to the control group. (**B**) Relative expression profiles of six immune-related genes in the hepatopancreas after ammonia stress. Statistical significance: * *p* < 0.05; ** *p* < 0.01; *** *p* < 0.001.

In the oxidative phosphorylation pathway, the core genes are predominantly associated with NADH dehydrogenase subunits (*Ndufb11*, *NuoB*, *Ndufs4*, *Ndufv2*, *Ndufa5*, etc.), cytochrome c oxidase subunits (*COX7A*, *COX11*, *COX6B*, *COX4*, etc.), and the mitochondrial ATP synthase complex (*ATP5MF*, *ATP6V1F*, *ATPeV0E*, etc.) (Figure 8A). Seven genes were randomly selected for validation of differential expression, showing upregulation of *Ndufs4*, *Ndufv2*, *Ndufa5*, *COX6A*, *ATP5MF*, and *ATPeV0E*, while *COX11* was downregulated (Figure 9A). Notably, *Ndufs4* and *ATPeV0E* exhibited upregulation by 2.13- and 2.25-fold, respectively, consistent with the KEGG bubble plot trends in the transcriptome. The upregulation of most genes is speculated to enhance ATP production through the oxidative phosphorylation pathway, maintaining immune efficiency. Six immune-related genes—*crustin*-2, *lectin*, *ALF3*, *chitinase 3*, *PPAF2*, and *CTSB*—were also randomly selected, with most showing downregulation by more than two-fold (Figure 9B). This downregulation likely contributes to the observed repression of the lysosomal pathway.

NADH dehydrogenase (ubiquinone) Fe-S protein 4 (*Ndufs4*), NADH dehydrogenase (ubiquinone) flavoprotein 2 (*Ndufv2*), and NADH dehydrogenase (ubiquinone) 1 alpha subcomplex subunit 5 (*Ndufa5*) are integral components of the NADH dehydrogenase family within mitochondrial respiratory chain complex I, the largest and primary complex responsible for electron transfer via the electron transport chain. These enzymes facilitate the transfer of electrons to FMN, oxidizing NADH to NAD^+^ while reducing FMN to FMNH_2_, ultimately transferring electrons to coenzyme Q (UQ), a process essential for cellular energy metabolism [84,85]. The COX complex plays a critical role in electron transfer within the oxidative phosphorylation process and is responsible for generating approximately 90% of the ATP required for muscle energy [86]. Cytochrome c oxidase assembly protein subunit 11 (*COX11*) and cytochrome c oxidase subunit 6A (*COX6A*) are key subunits of cytochrome c oxidase. The F-type H+-transporting ATPase subunit f (*ATP5MF*) and V-type proton ATPase subunit e-like (*ATPeV0E*) are localized within the mitochondrial inner membrane, facilitating ATP synthesis by promoting ADP phosphorylation under the proton gradient across the membrane [87].

In studies examining the impact of Tralopyril on zebrafish, the downregulation of genes such as *Ndufs4* suggested that TP impairs metabolism by disrupting mitochondrial function [88]. Similarly, research on the effects of nano-plastics on zebrafish intestines demonstrated that nano-plastic ingestion induced electron transfer dysfunction and inhibition of mitochondrial oxidative phosphorylation (OXPHOS), leading to *Ndufs4* downregulation [89]. In zebrafish embryos, *COX6A* expression was significantly upregulated after 24 h of PBDE exposure [90]. The seawater-domesticated fish subjected to low-temperature stress exhibit enhanced oxidative phosphorylation to increase energy production [91]. Tine et al. reported the overexpression of oxidative phosphorylation-related genes in the gill tissues of fish exposed to high salinity [92]. These findings align with the results of the current study, where the majority of mitochondrial oxidative phosphorylation core genes in *E. j. sinensis* were upregulated. It is hypothesized that ammonia stress in *E. j. sinensis* induces damage to the hepatopancreas and triggers oxidative stress, resulting in excessive ROS production. Mitochondria, being the primary site of ROS production, are particularly susceptible to ROS-induced damage. To mitigate this damage, the organism likely upregulates the oxidative phosphorylation pathway, generating substantial ATP to counteract cellular injury, maintain immune function, and bolster antioxidant capacity.

Antimicrobial peptides such as ALFs and crustins are critical components of the immune defense in crustaceans. The antimicrobial activity of *crustin-2* is mediated by its mature peptide region at the C-terminus. In *E. j. sinensis*, the *crustin-2* gene and its recombinant protein exhibit antibacterial properties, with potential applications in drug production, feed additives, preservatives, and as agents to extend product shelf life. Anti-lipopolysaccharide factor 3 (*ALF3*) and *Chitinase 3* play an immunomodulatory role, contributing to the immune response against pathogens. Phenoloxidase-activating factor 2-like (*PPAF2*) and *Lectin*, acting as model protein receptors in humoral immunity, provide protection against bacterial invasion and help maintain the body’s physiological integrity. Cathepsin B (*CTSB*), a key gene in the lysosomal pathway, is involved in immune regulation through its role in antigen processing and presentation, which aids in the initiation of immune responses. Additionally, *CTSB* influences the regulation of apoptosis by modulating the hydrolytic activity of apoptosis-related proteins.

Research has demonstrated that the expression levels of *crustin-2* and *lectin* mRNA in crayfish infected with White Spot Syndrome Virus (WSSV) are reduced [93]. Conversely, gene expression levels of anti-lipopolysaccharide factors (*ALFs*) and *crustin* in *Macrobrachium rosenbergii* are upregulated following baicalin treatment or *Vibrio parahaemolyticus* infection [94]. In studies where N-acetylcysteine (NAC) protects *E. j. sinensis* from T-2 toxin, the mRNA expression of antimicrobial peptides such as *ALF3* and *crustin-2* is significantly elevated [95]. Additionally, the expression of *CTSB* mRNA increases substantially in *Litopenaeus vannamei* after 120 h of starvation [96]. These trends in immune gene regulation within the lysosomal pathways across various crustacean tissues contrast with the findings of the current study. The observed downregulation of immune-related genes in this study may be attributed to oxidative damage induced by ammonia stress, which likely suppresses other metabolic pathways, leading to insufficient energy supply for lysosomal function. As a result, the organism may be unable to sustain its response to damage, causing a downregulation of lysosomal pathways, thereby impairing their associated immune functions.

## 4. Conclusions

This study identified a substantial number of DEGs through transcriptome analysis and examined the impact of ammonia stress on the immune and antioxidant functions of the hepatopancreas and hemolymph in *E. j. sinensis* via histopathology, TUNEL assays, and humoral enzyme activity measurements, offering insights into the crustacean immune system. After 96 h of ammonia stress, in order to maintain immune function and antioxidant capacity in response to hepatopancreatic damage caused by excessive ROS, the corresponding reaction mechanism was made. In contrast, the downregulation of immune-related genes and tissue damage appears to be linked to the suppression of the lysosomal pathway, impairing the organism’s ability to sustain its immune response. These findings contribute to a deeper understanding of energy metabolism and immune mechanisms in *E. j. sinensis* under ammonia stress and provide essential data for supporting the sustainable development of its industry.

## Figures and Tables

**Table 1:** Primer Names.

Primer Names	Sequences (5′–3′)
β-actin-F	GCATCCACGAGACCACTTACA
β-actin-R	CTCCTGCTTGCTGATCCACATC
Ndufs4-F	AATCTGGCACTCACAATC
Ndufs4-R	AAGCAATGGCATCCTC
Ndufv2-F	AAAACAACCCAGATACTTCC
Ndufv2-R	AGTTCCCGACCTGACG
Ndufa5-F	GATGCCCTCAGATGCT
Ndufa5-R	CCACTTCCACTGTCCCT
COX6A-F	CGCTCCATTTCCACCG
COX6A-R	CCACTAGCAGGGACACCAT
COX11-F	CACAGCACAGAGCGAGTC
COX11-R	CGCCCGCAGGTAAAGT
ATP5MF-F	GGAGAACTTGGTGCCT
ATP5MF-R	CATCTTGCCGTAGTTTAT
ATPeV0E-F	GATGACTGGTGGTGGG
ATPeV0E-R	GTATGGCGATGATGGTT
crustin-2-F	GCGACAGGAACCAGAAG
crustin-2-R	AAGCGTCACAGCAGCAC
Lectin-F	GGCGGCTGCTTCTACTT
Lectin-R	ACGTCCACGAACCCTCA
ALF3-F	TCTGGTCTATGGCACAACG
ALF3-R	AGTCCCGAGTGGCTTCC
chitinase 3-F	GCGAACCTCGACCTCAT
chitinase 3-R	TCCCTGGGCAATCTTTT
PPAF2-F	CCATCGGCTTCAACAAT
PPAF2-R	TGATGACGGGATTCTTACA
CTSB-F	CGGGCGGAACTTCAACA
CTSB-R	CGGTGCGGGAATCAAAC

**Table 2:** Quality of transcriptomic sequencing data of *Eriocheir japonica sinensis*.

Samples	Clean Reads	Clean Bases	GC Content	%≥Q30
Es-He-CK1	20,791,412	6,226,997,816	50.96%	95.57%
Es-He-CK2	19,925,204	5,968,030,201	51.08%	95.48%
Es-He-CK3	23,122,701	6,924,535,291	50.83%	95.34%
Es-He-3001	20,552,156	6,151,444,046	49.16%	95.15%
Es-He-3002	20,324,902	6,084,546,375	49.16%	95.74%
Es-He-3003	20,327,981	6,083,545,895	49.29%	95.75%

**Table 3:** Statistics of annotation success rates.

Annotation	Number of Genes	Percentage (%)
Annotated in NR	14,602	19.2
Annotated in SwissProt	7944	10.4
Annotated in PFAM	14,805	19.5
Annotated in KOG	10,175	13.4
Annotated in GO	14,180	18.6
Annotated in COG	4096	5.4
Annotated in KEGG	10,289	13.5
Total	76,091	100

## Data Availability

The original transcriptome data has been submitted to the NCBI database with the accession number PRJNA1108422.
